# An efficient filter with low memory usage for multimedia data of industrial Internet of Things

**DOI:** 10.7717/peerj-cs.589

**Published:** 2021-06-11

**Authors:** Parisa Goudarzi, Amir Masoud Rahmani

**Affiliations:** 1 Department of Computer Engineering, Islamic Azad University of Dezful, Dezful, Iran; 2 Future Technology Research Center, National Yunlin University of Science and Technology, Douliou, Yunlin, Taiwan

**Keywords:** Industrial Internet of Things, Efficient Two-Dimensional Filter, Multimedia data, Cuckoo filter, Memory efficiency, Quotient filter, Bloom filter, False positive rate, Chord architecture

## Abstract

One of the essential concerns of Internet of Things (IoT) is in industrial systems or data architecture to support the evolutions in transportation and logistics. Considering the Industrial IoT (IIoT) openness, the need for accessibility, availability, and searching of data has rapidly increased. The primary purpose of this research is to propose an Efficient Two-Dimensional Filter (ETDF) to store multimedia data of IIoT applications in a specific format to achieve faster response and dynamic updating. This filter consists of a two-dimensional array and a hash function integrated into a cuckoo filter for efficient use of memory. This study evaluates the scalability of the filter by increasing the number of requests from 10,000 to 100,000. To assess the performance of the proposed filter, we measure the parameters of access time and lookup message latency. The results show that the proposed filter improves the access time by 12%, compared to a Fast Two-Dimensional Filter (FTDF). Moreover, it improves memory usage by 20% compared to FTDF. Experiments indicate a better access time of the proposed filter compared to other filters (i.e., Bloom, quotient, cuckoo, and FTD filters). Insertion and deletion times are essential parameters in comparing filters, so they are also analyzed.

## Introduction

The industrial Internet of Things (IoT) consists of embedded smart devices that can collect and exchange data using digital technologies growing and expanding rapidly. As a result, IoT includes a wide range of different technologies such as Radio Frequency Identification (RFID) tags, communication protocols, data mining, and machine learning that enable users to query data. Objects have unique identifiers with unlike numeric or alphanumeric strings that can be used for various applications. Therefore, it is expected that many devices with network connectivity will be able to connect to Wireless Sensor Network (WSN), Wireless Local Area Network (WLAN), Mobile Ad Hoc Network (MANET), mobile networks, and the Internet. The IoT makes it possible to develop programs in various fields, such as healthcare (Silvano & Marcelino, 2020; Din et al., 2019; Garg et al., 2019).

Wireless Multimedia Sensor Networks (WMSNs) can receive multimedia data from the environment using low-cost hardware, such as Complementary Metal-Oxide-Semiconductor (CMOS) cameras. WMSNs can retrieve higher sensor information levels or richer information types, such as audio, video, and image. Thus they provide more accurate information about the environment. Sensors usually have directional sensing models in Wireless Directional Sensor Networks (WDSNs). Each sensor in a WDSN, including a camera sensor, radio sensor, ultrasound sensor, or radar sensor, has distinctive features. However, WDSNs have unique features, such as limited sensing angles, directional sensing, communication amplitude, and line-of-sight communication. Because of these features, the existing coverage control methods and theories of traditional omni-directional WSNs cannot be used for WDSNs (Akyildiz, Melodia & Chowdhury, 2007; Guvensan & Gokhan Yavuz, 2011).

If all intelligent objects (objects that use sensors or processors to communicate with users and computer systems) are collected in a centralized system, the system will not be scalable. As a result, one of the possible solutions for collecting, filtering, and integrating data is to use distributed systems. One of the most popular data structures in distributed systems is a Bloom Filter (BF), which is widely used in these systems. Reducing the management cost of huge multimedia data, security challenges, and resistance to various attacks have justified using BFs in heterogeneous multimedia data. Several types of BFs, such as Quotient Filter (QF), have an improved performance using a single hash function. Unlike a BF, the Cuckoo Filter (CF) can detect, insert, and display elements without increasing storage. Consequently, these excellent features justify the use of CFs in various applications (Ramezanian et al., 2020; Singh et al., 2018; Singh et al., 2020; Xiong et al., 2018).

In this article, a new type of Approximated Membership Query (AMQ) data structure for one of the industrial IoT applications called Efficient Two-Dimensional Filter (ETDF) is introduced. The proposed filter is a possible data structure that examines an element’s membership in a set by efficiently using the available space. A query for an element determines whether the element is not in the set or more likely is in the set. Deletion and fast query operations are possible in the proposed filter with a low false-positive rate. The proposed filter uses a hash function and is integrated into a CF to respond more quickly to requests. In general, the goals of the proposed filter are: easier access to elements with low false positives compared to BF, QF, and Fast Two- Dimensional Filter (FTDF), as well as lower memory usage, faster response to requests, and the capability of deletion.

The innovations of the present study are as follows:

• Proposing a hybrid efficient two-dimensional filter as the combination of FTDF and CF
• Adding a new column called value to the proposed filter structure to insert, query, and multimedia elements faster
• The higher performance of the proposed filter than the other filters due to the use of multimedia data
• Faster update of multimedia databases because the proposed filter supports deletion

The rest of the article consists of the following sections. In ‘Related Works’, related studies are reviewed. The proposed method is described in ‘The Proposed Filter’. The simulation results and evaluations are expressed in ‘Evaluation’, and finally, ‘Conclusion’ concludes the paper.

## Related works

In this section, the published related works are reviewed. Furthermore, each group is introduced in detail.

### Using filters in IoT applications

Using a BF, the authors (Jeong et al., 2019) proposed a secure storage service for IoT environments based on a provable data possession model. Their proposed scheme is not based on a public key, such as Rivest-Shamir-Adleman (RSA), bilinear mapping, and homomorphism. The advantage of their method is processing a large amount of data, which saves time.

The authors (Hao et al., 2019) proposed a control scheme with an access policy to fully attribute hidden for cloud-based IoT. They also designed a fuzzy attribute positioning mechanism based on the Bloom garbled filter. Their mechanism helps the authorized recipients effectively determine attributes, successfully decrypt an encrypted text, and do not get help from unauthorized recipients who can access information with valuable features in the encrypted text.

The authors (Cui et al., 2020) proposed a novel participatory filtering algorithm based on Time Correlation Coefficient (TCC) and K-means with Cuckoo Search (CSK-means), called (TCCF). TCCF can gather similar users (using the k-means clustering method) and design a time factor to resolve interest drift over time for faster and more accurate advice. Moreover, these authors proposed an effective and personalized model based on Preference Pattern (PTCCF) to improve the quality of TCCF.

The authors (Goudarzi, Tabatabaee Malazi & Ahmadi, 2016) presented the Khorramshahr architecture using IoT technology for inventory management based on Chord architecture. The main advantages of this study are scalability, fault tolerance, privacy, and improvement of the lookup process using BF and QF.

### Data structure of filters

In this subsection, the data structure is examined in the BF, QF, CF, and FTDF. The data structures of BF, QF, CF, and FTDF are basic algorithms (data structures). In addition, many papers have used these data structures. Consequently, some parameters of these four data structures are examined and compared with the proposed filter in Section Results.

#### The Bloom filter

Bloom filter was proposed by Bloom (1970) as a random data structure to investigate the presence of an element in a set. A BF can perform two basic insertion and query operations using an array of m-bit positions. In the insertion operation, the elements that have to be added are given to hash functions, K hash functions then determine the positions, and finally, the value of the positions are set to 1. During the query operation, the desired element in a set is specified using the hash functions, which give K array positions; if all bits at the positions are 1, the element may be in the set. The accuracy of BF depends on its size (m), the number of elements in the filter (n), and the number of hash functions (K) (Marandi et al., 2017; Jiang, Duan & Wang, 2018). Depending on the hash functions, BF allows false positives (i.e., an element is reported as a BF member, but it is not in it) while it uses storage space. The false-positive probability (P_f_) is a function of m, n, and k, which is calculated as follows:

(1) \begin{eqnarray*}{P}_{f}=(1-{e}^{(-k \left( \frac{n}{m} \right) )})^{K}\end{eqnarray*}

#### The quotient filter

The QF is a hash table that stores the b-bit fingerprint of the elements. This filter utilizes the quotient mechanism proposed in Knuth (1973). In this mechanism, each element’s fingerprint is divided into two parts: the q most significant bits and the r least significant bits. The quotient and remainder are extracted from q and r, respectively. The QF uses a quotient as a bucket index for storing the remainder. Mapping an element to a slot may lead to a collision when two different fingerprints are mapped to the same quotient (Geil, Farach-Colton & Owens, 2018).

#### The cuckoo filter

The CF can perform the usual data structure operations (insertion and query) and deletion, resizing and merging. An empty CF is a set of buckets. Each bucket can store K fingerprints. A typical CF uses two hash functions to generate keys and two buckets. If the CF fails to insert the key in the first bucket (when it already contains k keys), it will try to insert it in the second bucket. If the second bucket is also occupied, then the CF uses a particular function called a displacement operation to deal with such a situation (Gupta & Breitinger, 2015).

#### The fast two-dimensional filter

The FTDF includes a two-dimensional array that uses a single hash function and has a low false-positive rate. This filter is composed of an m*n matrix. All matrix bits are initially set to zero. This filter is similar to the QF in (Knuth, 1973), where a fingerprint is divided into two parts, i.e., the q most significant bits and the r least significant bits. This filter maps each element only to one position inside the matrix (Shubbar & Ahmadi, 2019). In order to insert an element in this filter, the element is given to the hash function to obtain the fingerprint. After applying the quotient technique to the fingerprint, the filter specifies the insertion position by considering the q and r bits; finally, the element is added to the specified position. In order to query for an element, it is fed to the hash function to get the fingerprint. Next, the quotient technique is applied to the fingerprint to obtain the q and r bits as previously mentioned.

## The proposed filter

In this section, the problem statement and the proposed filter data structure are examined.

### Problem statement

The set S = {x_1_, x_2_…x_n_} includes n elements that are inserted in the two-dimensional array **A**_(*m*)∗(*n*+1)_. In the proposed filter, regardless of the values of n and m (positions for storing), the fingerprint technique is used for all elements (x) of S to obtain q and r. Therefore, given the specified q and r, every a is inserted into the position (q, r) of the array **A**. If the desired position is already occupied, a CF is used to obtain a new position for inserting the element. In order to check whether x exists in S or not, the position specified in **A** is investigated considering q and r. If the answer is No, then the positions where the element may be located are examined using the CF. If they do not contain the desired element, then it is not in the set. Otherwise, the element may be in the set. Due to using a combination of quotient and cuckoo filter techniques, it is very improbable to have the same fingerprints for two different elements. As a result, the false positives of the proposed filter is much lower than those in other filters. Table 1 presents the parameters used in the proposed filter.

**Table 1:** Parameters of the proposed filter.

**Parameters**	**Description**
S = {x_i_} i={1, 2, …, m} k = {0, 1, …, n} n j	Indicate the array of input elements Indicate the number of elements Indicate the number of column in the CF Indicate columns in the CF Indicate the cell number of value column in the proposed filter
q	Indicate rows in the proposed filter
r	Indicate columns in the proposed filter
v	Indicate Value column in the proposed filter
f	Indicate fingerprint
p	The number of fingerprint bits
h_1_	Indicate hash function 1 in the CF
h_2_	Indicate hash function 2 in the CF
e	Indicate empty slots in bucket array
A(q,r)	Indicate a two-dimensional array
bucket [i]	Indicate cells in bucket array in the CF

### Proposed filter structure

In this subsection, the data structure of the proposed filter is analyzed. Then, we explain how to insert, query, and delete.

The proposed filter is formed as an **A**_(m)∗(n+1)_ matrix. One of the structural differences between the proposed filter and the FTDF proposed in (Shubbar & Ahmadi, 2019) is an additional column in the proposed filter. The columns in this filter are labeled from zero up to n. The zero column, i.e., the value column, contains zero or one value; zero (one) indicates that the row is empty (occupied). Moreover, the proposed filter is combined with the CF.

**Figure 1:**
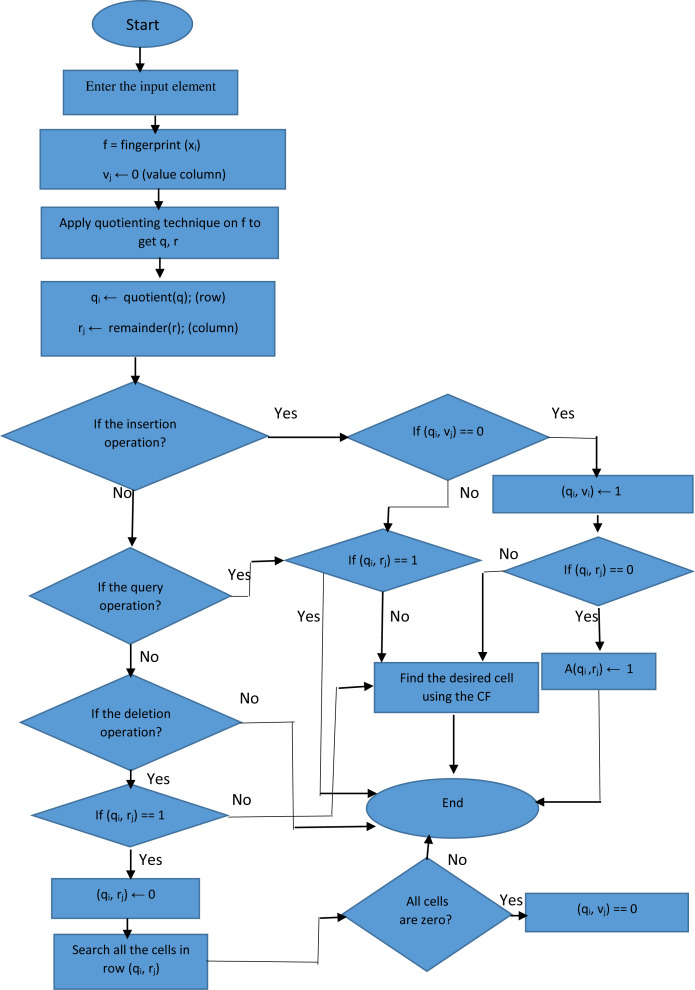
The flow chart of proposed filter.

All matrix bits are initially zero. The multimedia data that are meant to be included in the proposed filter data structure are first introduced to the hash function defined in the proposed filter. We use the Murmur hash function due to its high speed and easy implementation. The output of the hash function is a fingerprint, which is used to determine the position of an element in the proposed filter matrix. To specify the exact position for inserting the element using the fingerprint, it is necessary to determine the row and column in the two-dimensional structure of the proposed filter. For this reason, the input fingerprint of an element x, f_x_, is partitioned into r least-significant bits, f_x_^r^ = f_x_ mod 2 ^r^(remainder), and q (=p (p-bit fingerprint) – r) most-significant bits, f ${}_{\mathrm{x}}^{\varphi }\lfloor {\text{f}}_{\mathrm{x}}/{2}^{\mathrm{r}}\rfloor $ (quotient).

(2) \begin{eqnarray*}{f}_{x}^{r}={f}_{x}~mod~{2}^{r}(remainder)\end{eqnarray*}

(3) \begin{eqnarray*}q=(p \left( p-bit~fingerprint \right) -r)\end{eqnarray*}

(4) \begin{eqnarray*}Quotient={f}_{x}^{\psi }\lfloor {f}_{x}/2\rfloor \end{eqnarray*}

If the position in the array is empty, the desired element is inserted into that position. Otherwise, for uniform distribution in the array by key compression, the desired element is dynamically inserted into the first appropriate position that is determined by the hash functions of CF. We use Murmur and CRC hash functions as CF hash functions, which have special features of easy implementation and high speed. The proposed filter avoids loading duplicate elements due to its support for removing extra cells, which is an advantage over the CF. Moreover, in the proposed filter structure, a column called value is used to determine whether the rows in the proposed filter are full or empty. If a cell in the value column contains zero, the proposed filter deletes extra rows; this is an advantage over CF and reduces memory usage. Figure 1 indicates the flowchart of the proposed filter.

After inserting an element into this filter, the element is given to the hash function to obtain a fingerprint. Then, the quotient technique is applied to the fingerprint to obtain q and r; using q and r bits, the position of insertion to the filter is determined. The candidate bucket is calculated based on the x-fingerprint. If any of the fingerprints in these cells match the element’s fingerprint, the filter returns a response. Then, the corresponding value in the value column is turned into one, and the element will be inserted into position **A**_q_*_r_. The desired element is dynamically inserted into the first appropriate position that is determined by the hash functions of the CF. Algorithm 1 shows the element insertion in the proposed filter.

**Table:** 

Algorithm 1: Insert in Proposed Filter
1. // A is the matrix name;
2. **Input** x_i_ : element i;
3. **Output:** insert x_i_ in A
4. **begin**
5. fingerprint = hash function (x_i_);
6. f = fingerprint(x_i_);
7. v_j_← 0
8. Apply quotienting technique on f to get q, r
The number of fingerprint bits equal to p, f _x_^r^= f _x_mod 2 ^r^ (remainder), and it is q= (p (p-bit fingerprint) –r) most-significant bits, f _x_^Q^⌊f_x_∕2^r^⌋ (quotient), Number of bits in the remainder (r <p) equals r, Number of quotient equals: q = f –r;
9. q_i_← quotient(q);
10. r_j_← remainder(r);
11. **if** (q_i_, v_i_) = 0 then
12. (q_i_, v_i_) = 1;
13. A(q_i_,r_j_) ← 1;
14. **return** done
15. h_1_ = hash (x_i_)
16. h_2_ = h_1_^®^ hash (f)
17. **if** bucket [h_1_] or bucket [h_2_] has an empty slot (empty slots in an array are called entry, e, which are in the array bucket [h_1_] or bucket [h_2_] for elements to be inserted) **then**
18. add f to that bucket;
19. **return** done
20. i = randomly pick h_1_ or h_2_;
21. k ← 0;
22. **for** k=0; k<n; k++ **do**
23. randomly select an entry e from bucket (i);
24. swap f and the fingerprint stored in entry e;
25. i = i^®^ hash (f);
26. **if** bucket [i] has an empty entry then
27. add f to bucket [i];
28. **return** done;
29. **return** failure

The element is fed to the hash function to capture the fingerprint, which can be further used to query the element. Then, the quotient technique is applied to the fingerprint to obtain the q and r bits, as previously mentioned. Given the position **A**_q_*_r_, the corresponding value in the value column is checked. If this value is zero, the element does not exist. Otherwise, it is likely in the set. According to the CF, the positions with the possibility of having the desired element are searched. If there is a position with a value of zero, the element is not in the set. The query for the elements is shown in Algorithm 2.

**Table:** 

Algorithm 2: Query in Proposed Filter
1. **Input**: x_i_ : element i;
2. **Output:** whether there is x_i_ element in A or not
3. **begin**
4. fingerprint = hash function (x_i_);
5. f = fingerprint(x_i_);
6. v_j_← 0;
7. Apply quotienting technique on f to get q, r;
The number of fingerprint bits equal to p, f _x_^r^= f _x_mod 2 ^r^(remainder), and it is q= (p (p-bit fingerprint) –r) most-significant bits, f _x_^Q^⌊f_x_∕2^r^⌋ (quotient), Number of bits in the remainder (r <p) equals r, Number of quotient equals: q = f –r;
8. q_i_← quotient(q);
9. r_j_← remainder(r);
10. **if** (q_i_, v_j_)== 1 **then**
11. **if** A(q_i_ ; r_j_) == 1 **then**
12. **return** True;
13. **else**
14. h_1_= hash (x_i_);
15. h_2_= h_1_^®^ hash (f);
16. **if** bucket [h_1_] or bucket [h_2_] has f **then**
17. **return** True;
18. **return** False;
19. **return** False;

The proposed filter performs the deletion operation in the following steps: the quotient technique determines the row and column numbers, and the fingerprint is then retrieved. Next, the value at the position is changed from one to zero. If the content at that position is already zero, the positions with the possibility of having the desired element are searched using the CF. Next, the desired row is searched, and if all positions in that row are zero, the value corresponding to the row in the value column is changed to zero. Element deletion is shown in the following algorithm.

**Table:** 

**Figure 2:**
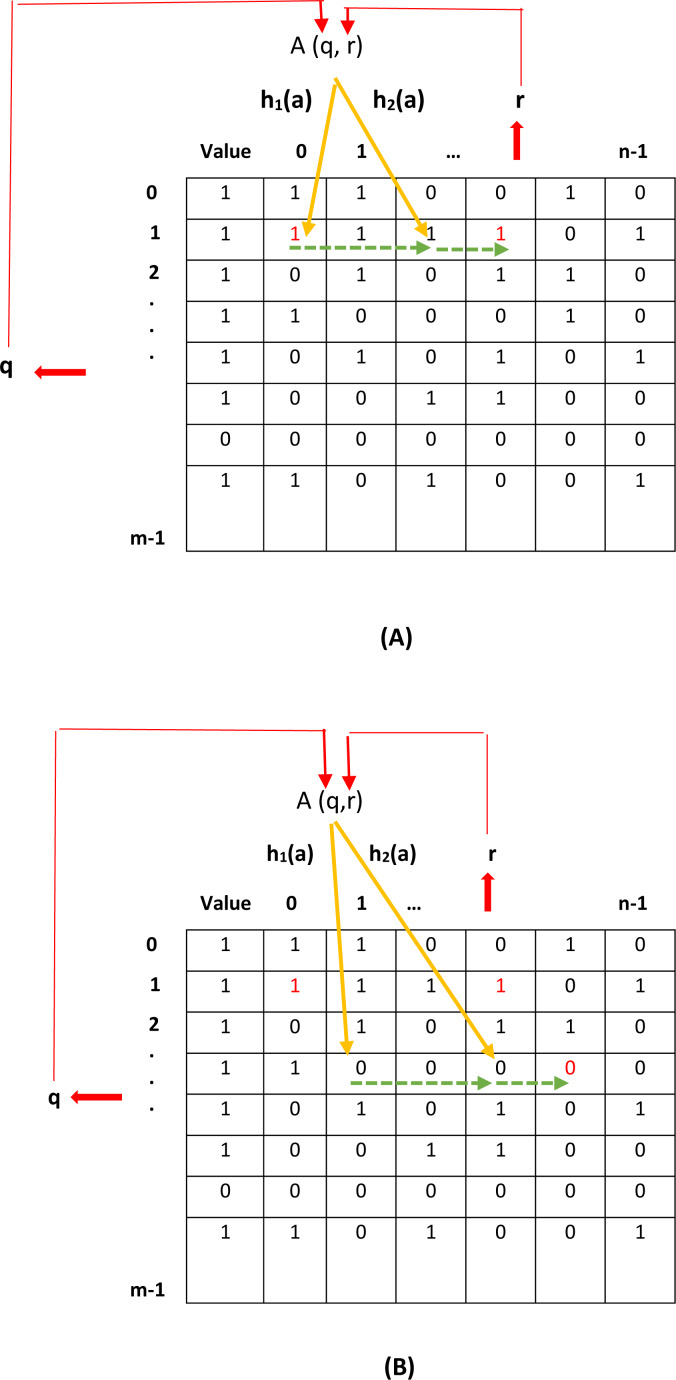
Add and remove an element in the proposed filter. (A) add an element, (B) remove an element.

Algorithm 3: Deletion operation
1. **Input:** x_i_: element i;
2. **Output:** Remove x_i_ from A
3. **begin**
4. fingerprint = hash function (x_i_);
5. f = fingerprint(x_i_);
6. v_j_← 0;
7. Apply quotienting technique on f to get q, r
The number of fingerprint bits equal to p, f _x_^r^= f _x_mod 2 ^r^(remainder), and it is q= (p (p-bit fingerprint) –r) most-significant bits, f _x_^Q^⌊f_x_∕2^r^⌋ (quotient), Number of bits in the remainder (r <p) equals r, Number of quotient equals: q = f –r;
8. q_i_← quotient(q);
9. r_j_← remainder(r);
10. **if** A(q_i_ , r_j_) = 1 **then**
11. A(q_i_; r_j_) ← 0;
12. **else**
13. h_1_= hash (x_i_);
14. h_2_= h_1_^®^ hash (f);
15. **if** bucket [h_1_] or bucket [h_2_] has f **then**
16. remove a copy of f from this bucket;
17. **return** True;
18. **return** False;
19. k ← 0;
20. **While** k <= n-1 **do**
21. **if** A(q_i_, k)== 0 **then**
22. k ←k+1;
23. **else**
24. break;
25. (q_i_, v_j_) ←0;

Figures 2A and 2B show, respectively, the insertion and deletion operations of elements in the proposed filter. In Fig. 2A, the location of inserting a new element is specified using q and r values. However, as shown in this figure, the location specified for inserting the new element is already occupied. Consequently, the new element is inserted into the first empty location specified by the CF (two hash functions defined in the CF). In Fig. 2B, the location of the element to be deleted is specified by q and r. However, as shown in this figure, the specified location is empty. Therefore, using the CF, other locations that may have stored the desired element need to be considered. Finally, the location of the desired element is found and its value is changed from one to zero.

### System description

The proposed filter is implemented on the architecture presented in (Goudarzi, Tabatabaee Malazi & Ahmadi, 2016). The pieces of information read by IoT sensors and cameras are sent to the computers in the warehouse and stored in the database (DHT). A user can search for goods information only through the DS in the application layer. Then, the searched information is converted into an ID in the ONS module in the application layer. The ONS in the application layer identifies the warehouses that can hold the searched goods and sends a request to the ONS in the relevant warehouse. Then, the DHT searches the relevant warehouse for product information, and by simultaneously using the physical layer, it traces the desired product in the warehouse. If the goods have been displaced or taken out of stock, the DHT in that warehouse updates its information and notifies the ONS in the warehouse. Finally, the search result is presented to the user. Khorramshahr architecture consists of a physical layer, middleware layer, and application layer.

**(A)** **Physical layer:** In the physical layer of the architecture presented in (Goudarzi, Tabatabaee Malazi & Ahmadi, 2016), only tags and readers are used to control the products/goods. There are several goods such as food and groceries that need to be constantly checked. As a result, cameras and WSDN sensors are needed to track these goods in the proposed architecture. Consequently, the physical layer of the proposed architecture includes RFID tags, Readers, WSDN sensors, and cameras.

**(B) Middleware layer:** There are two components (the middleware and the Distributed Hash Table (DHT) to store the goods information) in the middleware layer. The middleware is responsible for processing the information from readers and cameras. This means that it collects, filters, and prioritizes the incoming event streams from physical layer and generates the report by filtering, aggregating, and prioritizing the results and sending them to the DHT component. The second component of this layer is the DHT, which has the responsibility of organizing the goods types and the goods information from numerous warehouses.

**(C) Application layer:** There are two components (Object Name Service (ONS) and Discovery Service (DS)) in the application layer. The ONS is responsible for looking up the goods types from a Chord-based DHT. In the proposed design, the ONS is the service used to provide a list of warehouses in which a certain goods type can be stored. Moreover, if any warehouse needs information about goods in other warehouses, it should send a request to the ONS in the warehouse and then that request is sent to the ONS of the destination warehouse. In addition, the DS is defined in the application layer to search for goods inside and outside the port and to send requests from other (outside) warehouses.

**Figure 3:**
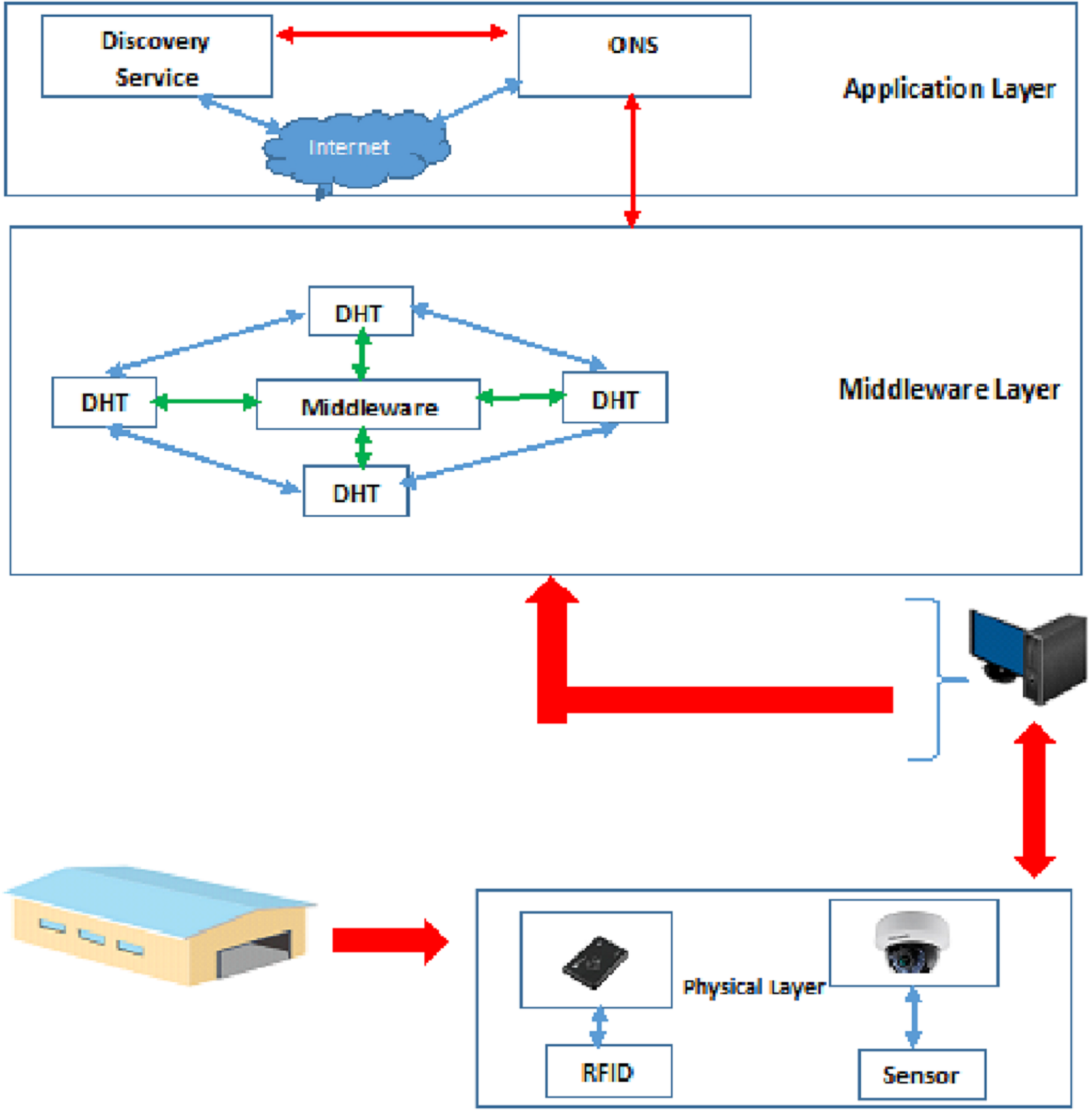
Proposed architecture in Goudarzi, Malazi Tabatabaee & Ahmadi (2016).

Two databases are defined in this architecture (DHT database in the middleware layer and ONS database in the application layer). Consequently, the authors use Double-Chord approach. Figure 3 shows the architecture proposed in (Goudarzi, Tabatabaee Malazi & Ahmadi, 2016).

Each of the warehouses is considered as a node. In each warehouse there is a computer that stores the data of the goods. In fact, the warehouses are considered as chord nodes. Getting data at these nodes engages at least two nodes for IoT operations. Goods data are collected using cameras and readers and sent to the computer in the warehouse. The displacement of goods (transfer to another warehouse) is reported to the computer in the warehouse. After updating its database, the DHT node delivers the updated information to the ONS (available in the same warehouse). The ONS provides updated information to other warehouses, so that the information of all goods to be updated at the port level. The ONS in the application layer contains information about the type of goods and the warehouses where they are stored. However, the DHT in the middleware layer only contains the product information (i.e., name, arrival date, expiration date, etc.). In general, the ONS in the application layer contains more details than the DHT in the middleware layer.

**Figure 4:**
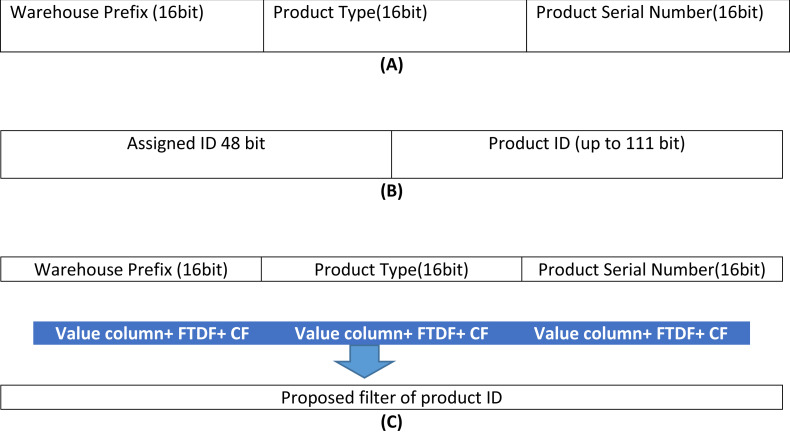
Building the proposed filter. (A) ID format for each product in the port, (B) the format of product ID, (C) the proposed filter.

According to the Khorramshahr architecture, an identification code (label/ID) is attached to each product. This ID has three sections. The first section is the warehouse prefix to indicate the warehouse ID (W) in which the product is stored. The second section is the product type (T). The product type is a unique ID in all the import/export warehouses and is based on the standard defined by the country. To access the product record in the node table, a 160 bits key is used. The achieved result for the assigned ID is depicted in Fig. 4.

## Evaluation

In this section, tools, evaluation scenarios, and simulation settings are introduced, and the measured parameters are then analyzed.

### Simulation tools and evaluation method

We used the 4.1 release of the OMNet++ platform. This platform was enhanced by INet Version 20101019 and OverSim 20101103.

The main purpose of this study is to evaluate the proposed filter, and analyze its performance and scalability by comparing with other filters, based on the data collected from one of the largest ports in Iran (Khorramshahr port). To analyze the scalability of the proposed filter, we increased the number of requests from 10,000 to 100,000. The number of terminals was changed from 100 to 500. We set the simulation time, Test TTL, and Ethernet channel delay to 18,000 s, 300 s, and 100 Mbps, respectively.

### Performance comparison

**Figure 5:**
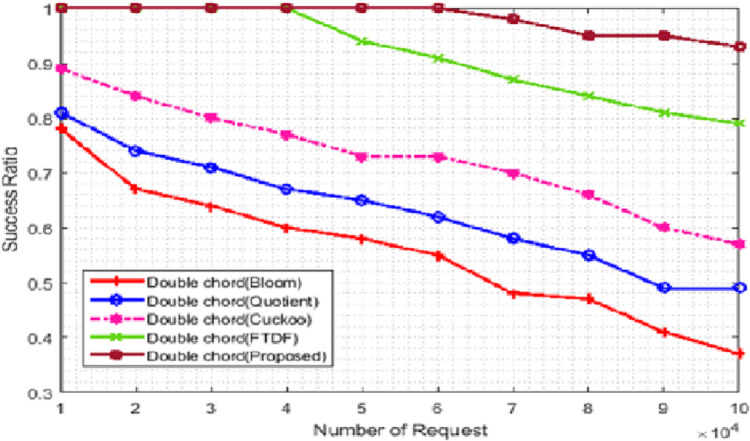
Success ratio (%) with different requests.

We compared the efficiency of the proposed filter with the success rate criteria for different types of filters. The number of terminals was set to 500. Figure 5 evaluate this metric. In Fig. 5, the *X*- and *Y*-axis represent the number of requests and the success rate, respectively. The success rate is equal to the number of requests with correct responses divided by the total number of requests multiplied by 100. The graph shows the responses to 50,000 requests, which are 100% and 98% for the proposed filter and the FTDF, respectively; that is, the proposed filter is faster in responding to the requests. As the number of requests increases, the performance gap becomes significantly large; for example, the corresponding values become 93% and 79% for 100,000 requests. When the number of requests increases, the requests move slower among the nodes, and they arrive at the destination node with some delay. As a result, the decrease in the success rate for BF, QF, CF, and FTDF is very noticeable. However, the proposed filter has a higher success rate compared to other filters, which indicates the more correct response to requests. For large amounts of data, the deletion speed of extra positions increases, so the search operation is slowed down.

From success rate, it can be concluded that the proposed filter has a higher efficiency compared to other filters in responding to requests correctly in IIoT applications such as warehouse management in ports. The basic network requirements of ports, which can be met by the proposed filter, include fast response to requests, fast access to goods data and sufficient goods data storage space. However, for large amounts of data, the deletion speed of extra positions increases, so the search operation is slowed down.

Table 2 compares the access time of the proposed filter with that of other filters. According to the success rate and latency parameters, the efficiency of the proposed filter has increased by 15%, 27%, 35%, and 42% compared to FTDF, CF, QF, and BF, respectively, so the proposed filter is more efficient than other filters.

**Table 2:** Comparing the access time of various filters.

**Access time Comparison**	FTDF	CF	QF	BF
Proposed Filter	15%	27%	35%	42%

#### False positive rate

**Figure 6:**
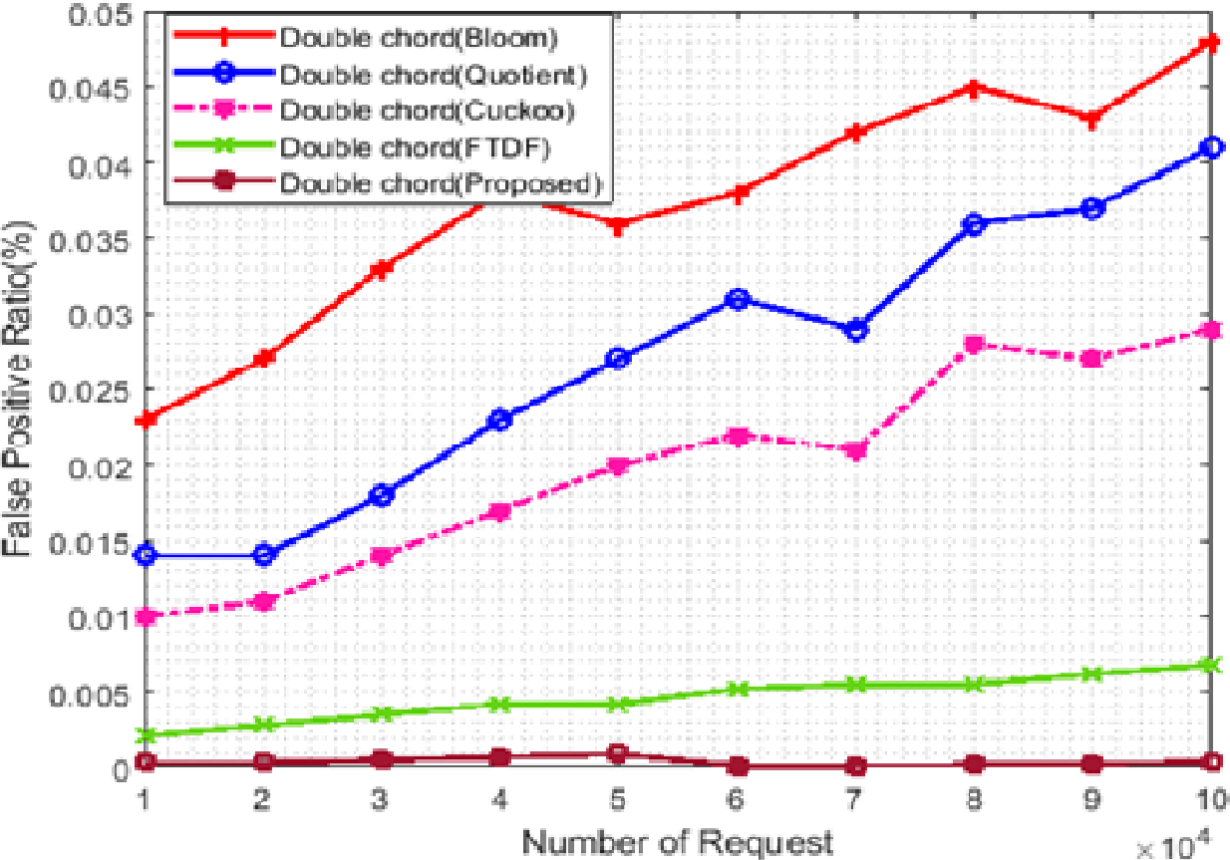
False positive rate with different requests.

BF, QF, CF, FTDF, and the proposed filter were evaluated concerning the scalability of the number of requests and the number of terminals. The false-positive rate was chosen as a metric to compare these filters used in Khorramshahr architecture to analyze the pros and cons of using these filters. In this scenario, the number of terminals was set to 500, while the number of received requests ranged from 10,000 to 100,000. Figure 6 shows the simulation results. In this figure, the horizontal and vertical axes show, respectively, the number of requests and the false-positive rate in percent. Due to similar structures of the proposed filter and FTDF, the false positive rates of these two filters are close to each other and almost the same (with a difference of less than 0.02). The correct response to requests in various IIoT applications, especially in ports, is very important. Due to its low false positive rate, the proposed filter can better meet the needs of port than other filters.

#### Memory usage

**Figure 7:**
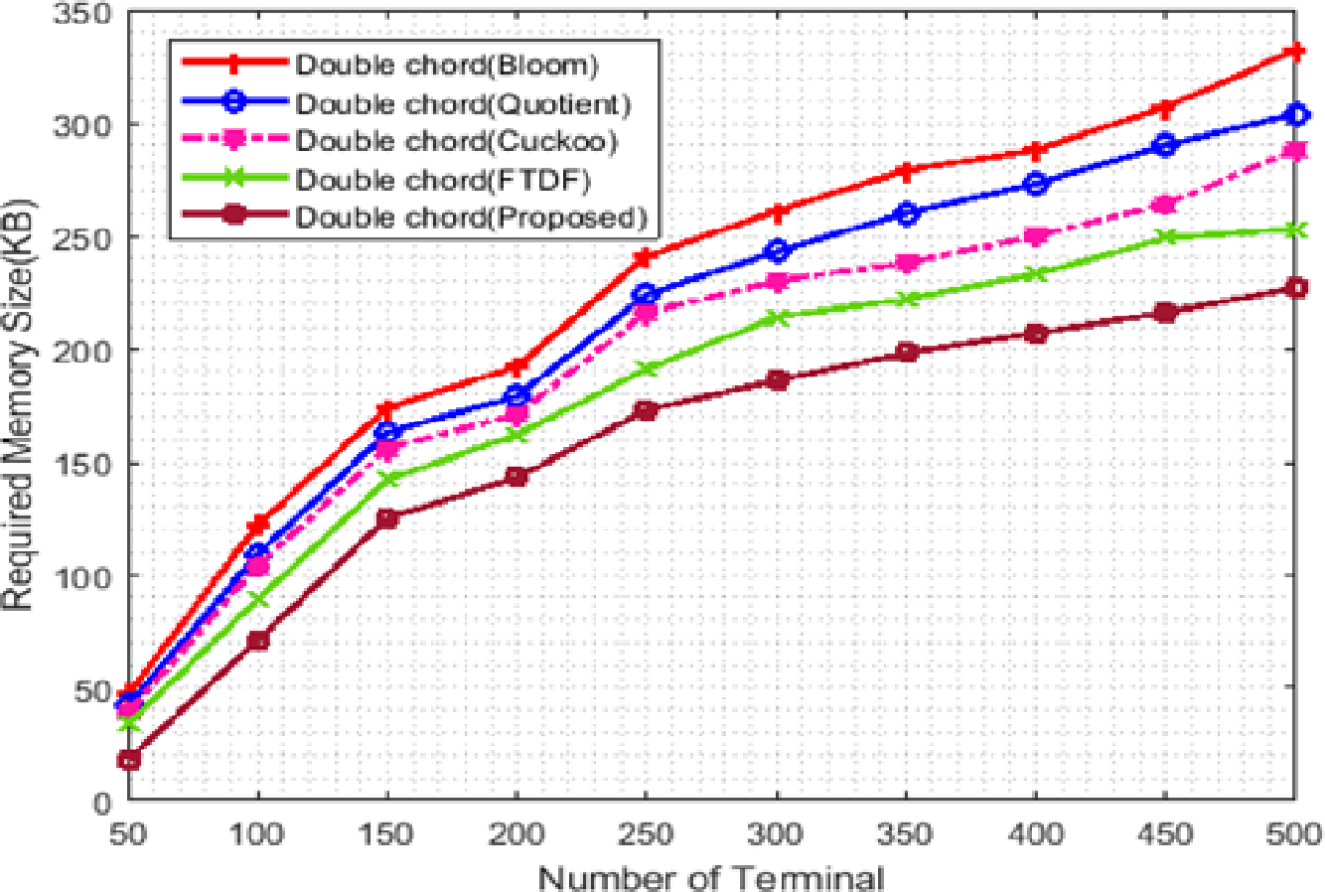
Memory consumption with different number of terminals.

Figure 7 shows the memory usage. The *X* -axis represents the number of terminals from 100 to 500, and the *Y* -axis shows the memory requirement. The double Chord for the proposed filter uses less memory than other filters. The value column defined in the proposed filter causes the rows that do not contain product information to be removed from the memory, reducing the memory size. The double Chord for the FTDF requires less memory than the other three filters. When the number of nodes increases, more requests are exchanged among nodes, the amount of memory usage increases, and the graph exhibits an ascending trend. The proposed filter improves memory usage compared to other filters. Due to the large amount of data available in IIoT applications, the memory level parameter is a very critical factor in these systems. The low memory usage of the proposed filter compared to other filters indicates the efficiency of the proposed filter in warehouse management (e.g., in ports).

#### Insertion time

**Figure 8:**
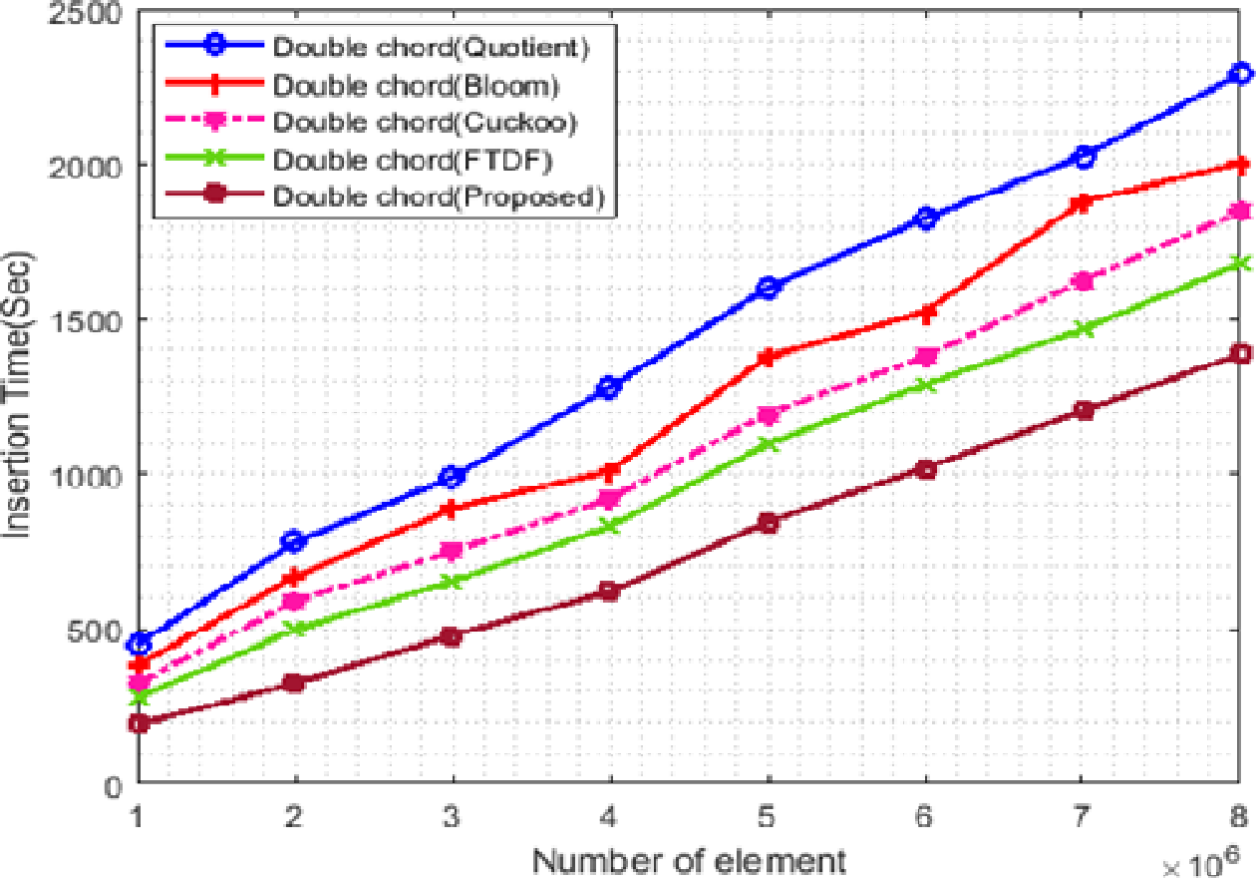
Insertion time with different elements.

Figure 8 shows the changes in insertion times of the filters. In this scenario, the number of terminals is set to 500 and the number of elements starts from 1 ∗ 10^6^ and increases to 8 ∗ 10^6^. Insertion into the proposed filter is faster than other filters. The proposed filter has less displacement to find the right position for insertion, so it requires less time compared to other filters. The QF has more overhead for insertion than the BF, which increases the insertion time of the QF.

#### Deletion time

**Figure 9:**
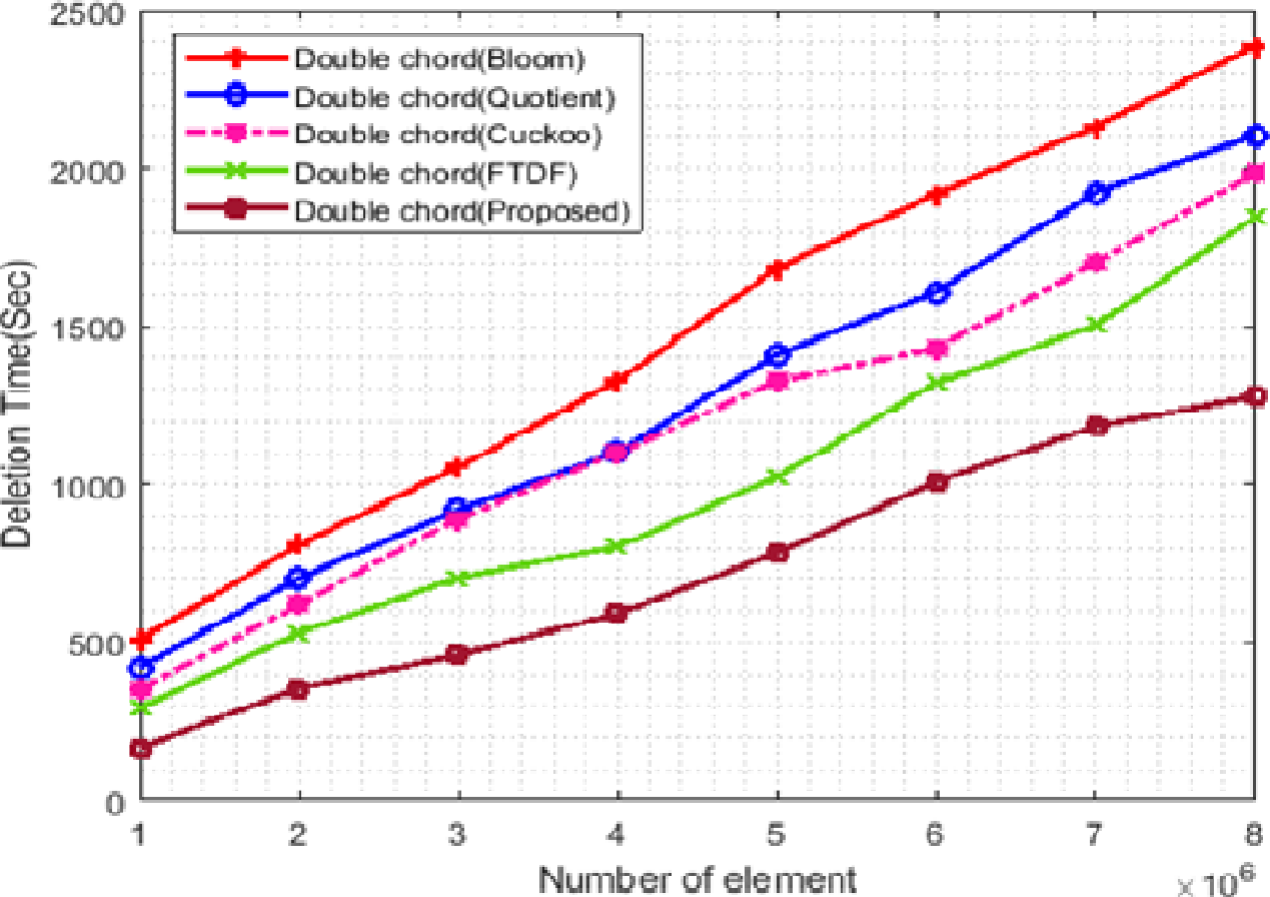
Deletion time with different elements.

Figure 9 shows the change in the deletion time of the filters. In this figure, the X- and *Y*-axis represent the number of elements and the deletion time, respectively. In this scenario, the number of terminals is set to 500, and the number of elements ranges from 1 ∗ 10^6^ to 8 ∗ 10^6^. Deletion in the proposed filter is done in a shorter time than other filters. The main reason for the shorter deletion time in the proposed filter is its hybrid structure. This structure uses q and r to search the cell of the desired element, and if it is empty, it will search the cells that may hold that item using the CF and delete the desired element. However, for deleting elements in the FTDF, only those cells are searched that are specified according to q and r. This will increase the deletion time and may even lead to failure in finding the element to delete.

BF lacks scalability for transport protocols, QF is highly inefficient in applications with non-flow data, FTDF Lacks scalability in multimedia applications, and CF has a low speed for multimedia applications due to reduced occupancy and linear reduction of false positives.

In general, the proposed filter supports deletion due to the use of a two-dimensional structure and the combination of FTDF and CF structures. Moreover, the proposed filter can insert and search faster a large number of requests compared to BF, CF, and QF. As a result, it can respond to more requests with lower false positive rates than other filters. In addition, the proposed filter can remove extra rows due to the use of an extra column compared to FTDF, and this improves memory consumption.

## Conclusion

We designed a new filter for IIoT applications, which reduces the amount access time. This new filter supports insert and delete operations with low false-positive rates and reduced memory usage. The test results showed that this filter performs more efficiently than other filters in managing warehouses data in ports. We also concluded that this filter can be used in applications such as data management in ports. As a result of this study, the new filter with appropriate speed and structure can meet network requirements in ports. The proposed filter can meet the basic network requirements of ports including fast response to requests, fast access to data, correct response to requests, and sufficient data storage space. However, for large amounts of data, the deletion speed of extra positions increases, so the search operation is slowed down.

**Limitations:** for a large number of requests, the query takes a longer time, so the response time increases, some requests are expired, and the overall performance of the proposed filter is adversely affected.

**Future works:** It is possible to study a new structure in which the proposed filter is combined with an artificial intelligence algorithm that can significantly increase the scalability and efficiency of the system in the case of a large amount of data.

##  Supplemental Information

10.7717/peerj-cs.589/supp-1 Supplemental Information 1 The program code, data, and license forms These datasets were used to evaluate different filters.

## Additional Information and Declarations

### Data Availability

The following information was supplied regarding data availability: The raw measurements, data, and code are available as a Supplemental File.
